# Survival time among patients who were diagnosed with tuberculosis, the precocious deaths and associated factors in southern Brazil

**DOI:** 10.1186/s41182-021-00320-4

**Published:** 2021-04-21

**Authors:** Danielle Talita dos Santos, Luiz Henrique Arroyo, Yan Mathias Alves, Luana Seles Alves, Thais Zamboni Berra, Juliane de Almeida Crispim, Josilene Dália Alves, Denisse Andrea Cartagena Ramos, Jonas Bodini Alonso, Ivaneliza Simionato de Assis, Antônio Vieira Ramos, Elma Mathias Dessunti, Ione Carvalho Pinto, Pedro Fredemir Palha, Ricardo Alexandre Arcêncio, Carla Nunes

**Affiliations:** 1 Ribeirão Preto College of Nursing (EERP/USP), University of São Paulo, Avenida dos Bandeirantes, 3900, Monte Alegre, Ribeirão Preto, São Paulo 14040-902 Brazil; 2 Postgraduate in the Public Health Nursing Program, Ribeirão Preto College of Nursing at University of São Paulo, Ribeirão Preto, São Paulo Brazil; 3 Inter-institucional Doctoral Program in Nursing, Ribeirão Preto College of Nursing at University of São Paulo, Ribeirão Preto, São Paulo Brazil; 4 Nursing Department, Federal University of Mato Grosso, Barra do Garças, Cuiabá, Brazil; 5 Facultad de Enfermería, Universidade Andrés Bello, Santiago, Chile; 6 University Center Dinâmica of Cataratas (UDC), Foz do Iguazu, Paraná Brazil; 7 State University of Londrina, Londrina, Brazil; 8 NOVA National School of Public Health, Universidade NOVA de Lisboa, Lisbon, Portugal

**Keywords:** Survival analysis, Tuberculosis, HIV, Mortality, Risk factors

## Abstract

**Background:**

A diagnosis of tuberculosis (TB) does not mean that the disease will be treated successfully, since death may occur even among those who are known to the health services. Here, we aimed to analyze patient survival time from the diagnosis of TB to death, precocious deaths, and associated factors in southern Brazil.

**Methods:**

We conducted a longitudinal study with patients who were diagnosed with TB and who died due to the disease between 2008 and 2015 in southern Brazil. The starting point for measuring survival time was the patient’s diagnosis date. Techniques for survival analysis were employed, including the Kaplan-Meier test and Cox’s regression. A mixed-effect model was applied for identifying the associated factors to precocious deaths. Hazard ratio (HR) and odds ratio (OR) with 95% confidence intervals (95% CI) were estimated. We defined *p* value <0.05 as statistically significant for all statistics applied.

**Results:**

One hundred forty-six patients were included in the survival analysis, observing a median survival time of 23.5 days. We observed that alcoholism (HR=1.55, 95% CI=1.04-2.30) and being male (HR=6.49, 95% CI=1.03-2.68) were associated with death. The chance of precocious death within 60 days was 10.48 times greater than the chance of early death within 30 days.

**Conclusion:**

Most of the deaths occurred within 2 months after the diagnosis, during the intensive phase of the treatment. The use of alcohol and gender were associated with death, revealing inequality between men and women. This study advanced knowledge regarding the vulnerability associated with mortality. These findings must be addressed to fill a gap in the care cascades for active TB and ensure equity in health.

**Supplementary Information:**

The online version contains supplementary material available at 10.1186/s41182-021-00320-4.

## Background

Even though the treatment of tuberculosis (TB) has been established since the late 1940s, worldwide, TB is one of the top 10 causes of death and the leading cause from a single infectious agent (*Mycobacterium tuberculosis*) [1]. In 2019, a total of 1.4 million HIV (human immunodeficiency virus)-negative people, in addition to some 300,000 people living with HIV/AIDS (PLWHA), died as a result of TB [1]. At present, a group of 30 countries accounts for 87% of TB cases worldwide, and Brazil is currently in 19th place in this world ranking [1], where the incidence of TB in 2019 was 36 cases/100,000 inhabitants, and the mortality was 2.2 deaths/100,000 people [2].

The geographical distribution of TB deaths is marked by inequalities and regional heterogeneities in the country, which ranges from 1.3 deaths/100,000 in the central-west region to 2.5 deaths/100,000 in the north [3]. The differences are more pronounced when considering the Brazilian capitals [3]. Mortality due to TB is strongly associated with vulnerabilities and social inequalities, and understanding this dimension of the problem is considered a fundamental strategy to elaborate public policy, advance equity, and mitigate the suffering occasioned by the disease, mainly in the population at risk [4].

The literature has evidenced the factors associated with TB death such as age, being male, coinfection HIV, diabetes mellitus, alcohol consumption, and tobacco smoking [5–7]. Vulnerability has also been identified with regard to lower educational level and income, socio-economic factors, location of one’s abode, unemployment, informal work, and homelessness [5, 8]. The association of TB deaths with failures in treatment has been investigated; failures occur mainly because of the side effects of drugs, vulnerabilities, non-inclusive and/or unfriendly policies of health services, non-unique therapeutic projects, and the absence of a link to health professionals [9].

Although these factors are well-known in the literature, we did not observe a distinction among the studies in relation to the time in which death occurred [4], as if the associated factors were independent of time. TB deaths that occur with a known diagnosis are serious and indicate that, at some stage of the care cascade, a gap existed that needs to be fixed to ensure the continuity of treatment, successful treatment, and adequate quality of care [10]. Thus, estimating how long patients survive and specifically when they died during treatment, and if this death was precocious (the first 60 days after diagnosis [11, 12]), may contribute to improving care and advancing the End TB strategy for disease elimination.

Although studying the survival time of patients and associated causes is crucial for improving care and surveillance, in practice, few studies have been performed on this issue [4], mainly in Brazil where the number of TB deaths is still high. Patients dying in the first stage of treatment (intensive phase), i.e., a precocious death, is unjustifiable and conflicting from a quality of care perspective, since the expectation is that patients will show progressive improvement in their health condition. Here, we aimed to analyze patient survival time from the diagnosis of TB to death, precocious deaths, and associated factors in southern Brazil.

## Methods

### Study design

This was a longitudinal study that followed TB patient survival, from the diagnosis date to the date of death through secondary data.

### Settings

The study was conducted in Curitiba, the capital of the State of Paraná, with an estimated population of 1,971,185 people and a demographic density of 4027.04 people per square kilometer [13]. This is a Brazilian state capital with a Human Development Index (HDI) of 0.823, placing Curitiba in tenth place in the national ranking. The percentage of people considered to be poor is at 1.73%, while 7.93% of the population is vulnerable to poverty; the GINI Index score is 0.55 [14]. The municipality of Curitiba had the following coefficients: prevalence of 14 cases per 100,000 people and mortality of 1.2 per 100,000 people [15]. The deaths were clustered in the southern region of the municipality and were associated with low HDIs in the respective regions [16].

### Participants, inclusion, and exclusion criteria

The participants of the study were patients with a diagnosis of TB, including cases of HIV coinfection, and who died due to disease between 2008 and 2015 considering causes A15.0 to 19.0 and B20.0 according to the International Classification of Diseases and Related Health Problems 10 (ICD-10). The criteria defined for diagnosis in Brazil are based on the recommendations of the Ministry of Health, which include medical history, physical examination, microbiological examination (sputum or some other appropriate sample) through microscopy or GeneXpert MTB/RIF assay (when available), and culture [17]. The certification of the cause of death is based mainly on a clinical evaluation by a physician who was following the patient or necropsy when patients died without medical assistance or in cases where the physician who provided assistance was unable to establish a diagnosis of the cause of death [18].

The basic treatment of sensitive TB (when the patient is not resistant to any of these drugs) was performed with rifampicin, isoniazid, pyrazinamide, and ethambutol for at least 6 months. If the patient was resistant to one of these drugs, the therapeutic regimen was changed and the treatment time was increased to 18 months [17]. We excluded patients when there was no record of the dates of diagnosis and death. Survival analysis was performed considering the difference between these dates.

### Data source and measurement

Data were obtained from the Mortality Information System*—Sistema de Informação sobre Mortalidade* (SIM), and the Notifiable Diseases Information System—*Sistema de Informação de Agravos de Notificação* (SINAN) through the Secretariat of Health of the State of Paraná (SESA). We estimated a patient’s survival time considering the total time (in days) that elapsed between the date on which the diagnosis of TB was confirmed and the date of death as a result of TB. Once we estimated the survival time, in sequence, we also identified the precocious deaths and associated factors. All patients were considered to estimate the survival time.

### Quantitative and qualitative variables under study

Table 1 shows all independent variables considered for the study. These variables organized according to socio-demographic characteristics, comorbidities, and information about the diagnosis and treatment of the patients; their classes are also described.

**Table 1:** Independent variables under study

Categories	Independent variables	Classes
Socio-demographic characteristics^a^	Gender	Female
		Male
	Age	Continuous
	Ethnicity	White/oriental
		Afrodescendant
	Educational level	8 years of schooling or more
		7 years of schooling or less
	Marital status	Married/common-law marriage
		Single/widowed/separated or divorced
Comorbidities^b^	Type of entry	New case
		Re-entry or retreatment
	Institutionalized	No
		Yes
	Examination: X-ray	Normal
		Yes, suspicious results
	Clinical category	Pulmonary
		Extrapulmonary
	Aggravation—use of alcohol	No
		Yes
	Aggravation—*diabetes mellitus* (DM)	No
		Yes
Information of the diagnosis and treatment^b^	Examination: bacilloscopy	Negative
		Positive
	Culture	Negative
		Positive
	Medication used: rifampicin	Yes
		No
	Medication used: isoniazid	Yes
		No
	Medication used: pyrazinamide	Yes
		No
	Medication used: ethambutol	Yes
		No
	Medication used: streptomycin	Yes
		No
	Medication used: other drugs (second-line TB drugs)	Yes
		No
	Supervised treatment—DOTS	Yes
		No

^a^Variables from the first database Mortality Information System *Sistema de Informação sobre Mortalidade* (*SIM*)

^b^Variables from the second database *Sistema de Informação de Agravos de Notificação* (*SINAN*)

### Data linkage

A record linkage was necessary to join the SINAN and the SIM considering a deterministic and probabilistic method [19]. The first step of the linkage was performed from the identification of the common identifier to both systems, which was the date of diagnosis and mother’s name of the patients. In the first step, we used the functions (PROCV) provided in the software Microsoft® Office Excel 2016.

For unpaired records in this phase, in accordance with literature [19], a probabilistic linkage using automated procedures was applied in order to identify the probability of a pair of records belonging to the same individual. Each death case identified in the SIM was matched to its respective case in the SINAN. The linkage was necessary for more consistency and completeness of data from patients followed up in the study. The SIM has more current and reliable information about the deaths than the SINAN, and many cases the SINAN was out of date regarding the death record and the dates were missing, which is relevant to estimating survival time. The date of diagnosis, clinical information, and treatment were variables contained only in the SINAN. All probabilistic step processing was performed using R Studio® version 4.0.3.

### Statistical methods

We applied descriptive statistics to obtain the absolute values and percentage frequencies of the categorical variables. In the case of continuous variables (time in days and age), we obtained the minimum and maximum values, arithmetic mean, median, and standard deviation (SD). The statistical analysis was performed in two phases and with different objectives. In the first one, with the objective of analyzing the survival time of patients and associated factors, we implemented the Cox proportional hazards regression model [20–22]. A descriptive analysis was carried out and the survival time was estimated for each factor under study, which were selected by statistical and clinical criteria. We elaborated the Kaplan-Meier plot for these factors [23], and in the survival analysis no outcome was censored, since the information of deaths due to TB was available for all study patients. The survival analysis was used to estimate the survival time among all patients under investigation.

Before introducing the variables into the model, we verified multicollinearity using the variance inflation factor (VIF) as the diagnostic criterion [24]. In order to assess the interaction effect between the exploratory variable considered in this phase, interaction plots were constructed for each pair of independent variables. Interaction effects occur when an independent variable, beyond affecting the dependent variable, also influences the value of another independent variable, which requires a more complex and robust analysis [25]. In the analysis, the occurrence of non-parallel lines in the respective plots was considered as the existence of an interaction [26].

Subsequently, we elaborated the Cox proportional hazards regression model with the associated factors [26] with and without interactions. To verify the consistency of the model and the non-violation of assumptions about the analysis performed, we evaluated the proportional hazard for the regression model with the chi-squared goodness of fit, following Schoenfeld [27]. Finally, we performed an analysis of the model residuals and the quality of the final adjustment [27]. Hazard ratio (HR) was calculated with 95% confidence intervals.

In the second phase, a mixed-effects model (mixed model) [28] was used to identify the factors associated with precocious deaths. Precocious deaths were categorized as yes or no, considering specifically two periods, i.e., 30 or 60 days counted from the diagnosis date.

The independent variables of fixed effects were type of entry (new case, relapse), TB-HIV coinfection (yes, no), alcoholism (yes, no), gender (male, female), age (years), and clinical form (pulmonary, extrapulmonary). For a random effect related to the patients, we assumed a normal distribution with a mean of 0 and constant variance.

Regarding the second phase, a generalized additive model for location, scale, and shape (GAMLSS) model was selected, because this model assumes that observations yi for *i* = 1,..., *n* with the conditional probability density function f (yi | θi) over θionde θi = (θi1,... θip) is a vector of parameters of size *p*, each one related to a set of independent variables. It also assumes a monotonous relation function, given by *g* (.), which relates the *k*th parameter θk with the exploratory variables of the model [29, 30].

In this analysis, a maximum of four parameters was required (*p* = 4), usually characterized by position (θ1 = *μ*), scale (θ2 = *σ*), asymmetry (θ3 = *υ*), and kurtosis (θ4 = *τ*). While the first two population parameters, represented by *μ* and *σ*, are referred to in the literature by position (or location) and scale parameters, respectively, the last two *υ* and *τ* are called shape parameters. According to this assumption, we have the following models [31]:

$$ \left.\begin{array}{ll}\mathrm{Parameter}\mathrm{s}\ \mathrm{of}\ \mathrm{position}\ \mathrm{and}\ \mathrm{scale}& \left\{\begin{array}{c}{g}_1\left(\mu \right)={n}_1={X}_1{\beta}_1+{\Sigma}_{j=1}^{J_1}{Z}_{j1}{\gamma}_{j1},\\ {}{g}_2\ \left(\sigma \right)={n}_2={X}_2{\beta}_2+\sum \limits_{j=1}^{J_2}{Z}_{j2}{\gamma}_{j2,}\end{array}\right.\\ {}\mathrm{Parameter}\ \mathrm{of}\ \mathrm{form}& \left\{\begin{array}{c}{g}_3\left(\nu \right)={n}_3={X}_3{\beta}_3+{\Sigma}_{j=1}^{j_3}{Z}_{j_3}{\gamma}_{j_3,}\\ {}{g}_4\left(\tau \right)={n}_4={X}_4{\beta}_4+{\Sigma}_{j=1}^{J_4}{Z}_{j4}{\gamma}_{j4}\end{array}\right.\end{array}\right\} $$

where *μ*, *σ*, *υ*, *τ*, and *xjk* for *j* = 1,…, *Jk* ek = 1,2,3,4 are vectors of length *n*, βk = (β1k,… βJ′kk) is a vector of size parameters J′k, *Xk* and *Zk* are matrices of fixed, known independent variables and of orders *n x J′k* and *n x qjk*, respectively. Finally, *γjk* is a *qjk*-dimensional random variable [31]. For all parameters of the final model, the odds ratio (OR) was calculated with its respective 95% confidence interval. We defined *p* value <0.05 as statistically significant for all statistics employed. The power of the final models obtained from the adjustments was calculated through Monte Carlo simulations. Data analysis was performed using the program R version 4.0.1.

## Results

### Participants

**Fig. 1:**
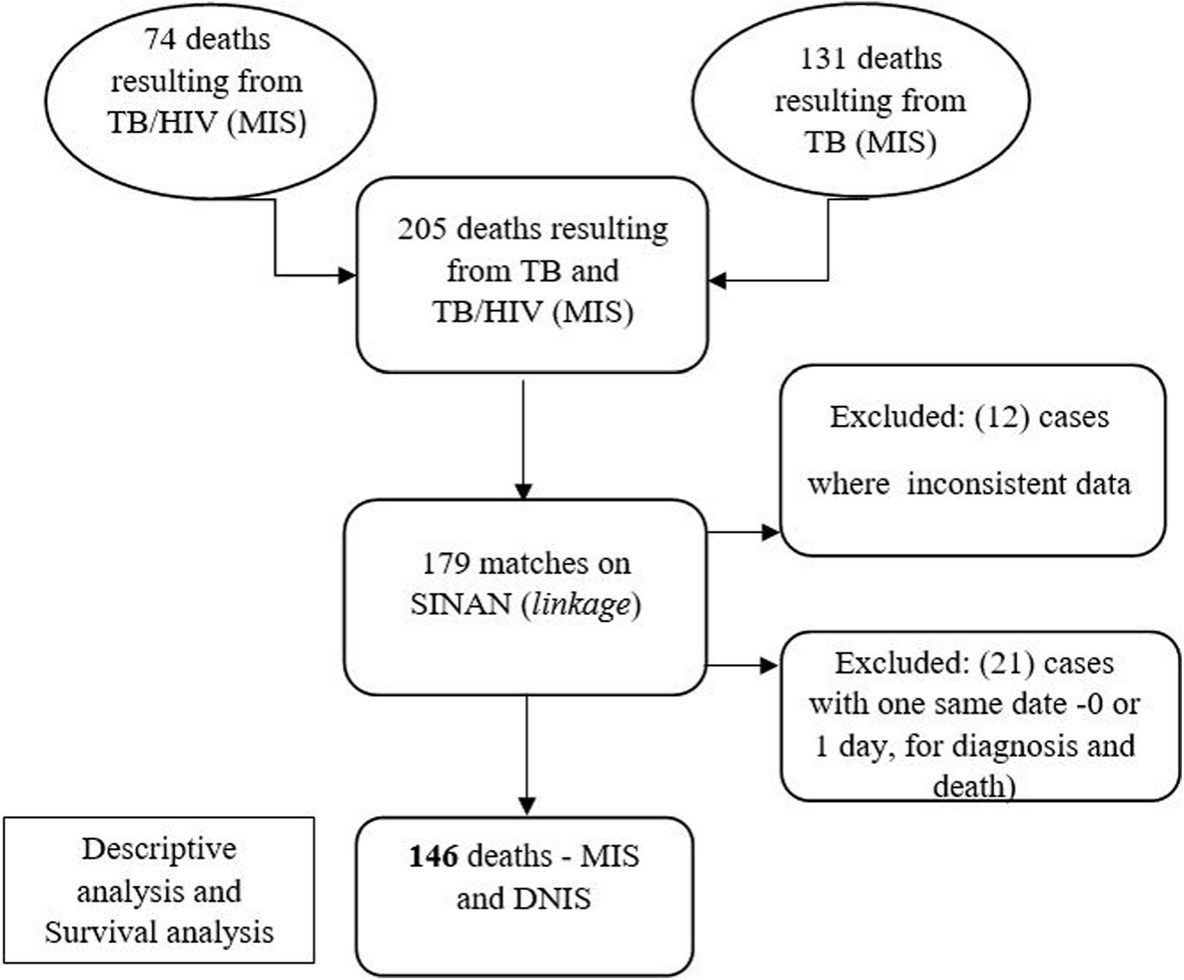
Flow chart of deaths analyzed in the study

A total of 205 cases were identified, and when the record linkage between the SIM and the SINAN was applied, 179 cases (87.3%) were potentially eligible. After application of the inclusion and exclusion criteria, including a high enough level of information that allowed follow-up of the patients retrospectively, a total of 146 patients were considered for the study. In Fig. 1, we show the flow chart presenting the number of individuals at each stage of the study.

### Descriptive data

Among the participants of the study, 84 (57.5%) had a diagnosis of TB and 62 (42.5%) were diagnosed with TB/HIV coinfection. The descriptive results are presented in Table 2, in which 114 (78.1%) were male. The white race was most prevalent (96 cases; 65.8%), and the mean age was 47 (minimum 20, maximum 83, median = 46, and SD = 14). The most common clinical presentation was pulmonary TB, with 106 cases (72.6%). There were in total 122 new cases (83.6%) and, in 124 of the cases (84.9%), the diagnosis was confirmed by radiographic examination. In Table 2, we also can observe the proportion of the variables that were not completed satisfactorily in the SINAN, indicated as “not reported” in the Table 2.

**Table 2:** Characteristics of the patients who died due to TB, Curitiba-Brazil

Variables	Categories	All deaths	
		***N*** (146)	(%)
Coinfection (basic cause)^a^	Yes (TB/HIV)	62	42.5
	No (only TB)	84	57.5
Gender (14)^a^	Female	32	21.9
	Male	114	78.1
Ethnicity (133)^a^	White or oriental	96	65.8
	Afrodescendant	40	27.4
	Not reported	10	6.8
Years of schooling^a^	0-7 years of schooling	84	57,5
	8 or more years of schooling	42	28,8
	Not reported	20	13.7
Marital status^a^	Married/common-law marriage	46	31.5
	Single/widowed/separated or divorced	84	57.5
	Not reported	16	11.0
Type of entry^b^	New case	122	83.6
	Re-entry or retreatment	24	16.4
	Not reported	0	0
Inpatient treatment^b^	No	115	78.8
	Yes	16	11.0
	Not reported	15	10.2
X-ray confirmation of diagnosis^b^	No	22	15.1
	Yes/suspicious	124	84.9
Clinical form^b^	Pulmonary	106	72.6
	Extrapulmonary	40	27.4
Alcoholism^b^	No	90	61.7
	Yes	50	34.2
	Not reported	06	4.1
Diabetes mellitus (DM)^b^	No	136	93.1
	Yes	03	2.1
	Not reported	07	4.8
Microscopy^b^	Negative	40	27.4
	Positive	81	55.5
	Not reported	25	17.1
Sputum culture (40)^b^	Negative	20	13.7
	Positive	20	13.7
	Not reported	106	72.6
Rifampicin^b^	No	06	4.1
	Yes	128	87.7
	Not reported	12	8.2
Isoniazid^b^	No	06	4.1
	Yes	128	87.7
	Not reported	12	8.2
Pyrazinamide^b^	No	06	4.1
	Yes	128	87.7
	Not reported	12	8.2
Ethambutol^b^	No	37	25.3
	Yes	97	66.5
	Not reported	12	8.2
Streptomycin^b^	No	131	89.7
	Yes	03	2.1
	Not reported	12	8.2
Ethionamide^b^	No	132	90.4
	Yes	01	0.7
	Not reported	13	8.9
Medication used: other drugs^b^	No	122	83.6
	Yes	07	4.8
	Not reported	17	11.6
Directly observed therapy (DOT)^b^	No	18	12.3
	Yes	114	78.1
	Not reported	14	9.6

^a^Variables from the first database Mortality information system *Sistema de Informação sobre Mortalidade* (*SIM*)

^b^Variables from the second database *Sistema de Informação de Agravos de Notificação* (*SINAN*)

### Statistics from survival analysis

The median survival from diagnosis to death was 23.5 days, with a minimum survival time of 2 days and maximum of 1688 days; the SD was 174.6 days and the mean was 73 days. The survival time according to each factor considered for the Cox proportional hazards is shown in Table 3. We found that the relapse cases were those with the highest average survival time; however, they were also the cases with the greatest dispersion in the studied population. Among the new cases, the survival time was 47.89 days and PLWHA had a longer survival time when compared to those without HIV infection. Additionally, patients with the extrapulmonary form presented a lower survival time when compared to those with pulmonary TB.

**Table 3:** Survival time according to each factor considered for the Cox proportional hazards regression model, Curitiba-Brazil

Explanatory variables	*N*	Mean of survival time (days)	Standard deviation of survival time (days)	First quartile of survival time (Q_1_)	Second quartile of survival time (Q_2_)	Third quartile of survival time (Q_3_)	Maximum survival time
Type of entry							
New case	122	47.89	67.83	7.00	21.00	60.25	349
Relapse	24	200.96	384.39	20.75	80.50	127.50	1688
TB/HIV coinfection							
No	84	48.44	79.66	7.00	21.00	58.75	513
Yes	62	106.39	248.89	9.25	36.50	100.50	1688
Alcoholism							
No	90	70.40	123.24	9.25	27.00	87.50	1008
Yes	50	78.92	246.31	7.25	23.50	49.75	1688
Gender							
Female	32	69.72	114.64	7.75	21.00	65.75	513
Male	114	73.98	188.55	8.00	25.00	81.00	1688
Clinical form							
Pulmonary	106	77.01	199.48	7.25	23.50	77.00	1688
Extrapulmonary	40	62.55	78.55	8.75	24.50	91.25	310

When the factors were considered according to quartiles, in the first quartile, the variables mostly had 7 to 9 days of survival time. The second quartile, which represents 50% of the sample, ranged from 21 to 80.50, with the majority remaining between 21 and 27 days.

**Fig. 2:**
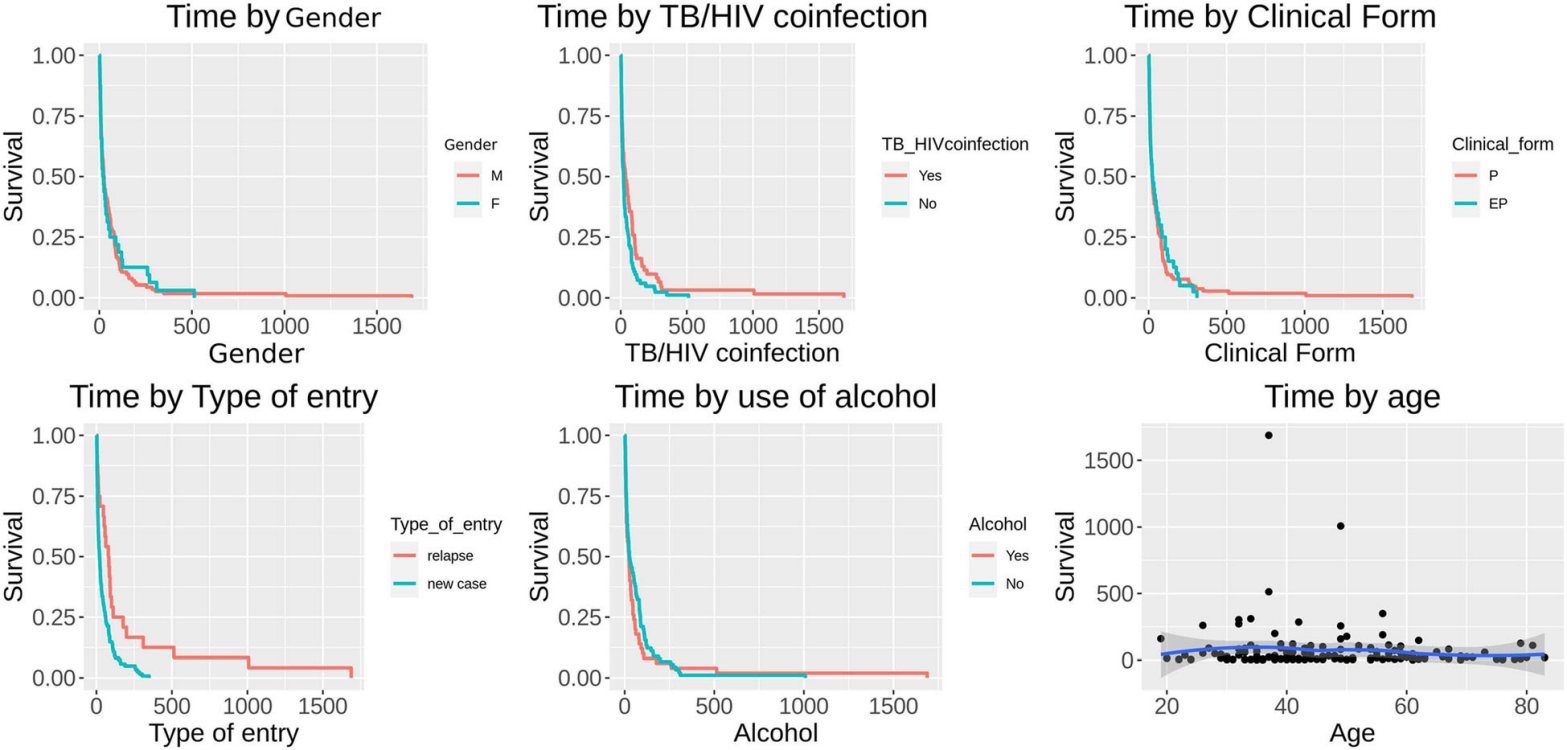
Curves obtained from the Kaplan-Meier survival analysis of the explanatory variables in patients who died of TB, Curitiba-Brazil. *Legend*: *dispersion time according to age and survival*

Figure 2 shows the curves obtained through the Kaplan-Meier survival analysis of the factors selected for the Cox model and the survival time of the continuous variable age.

**Fig. 3:**
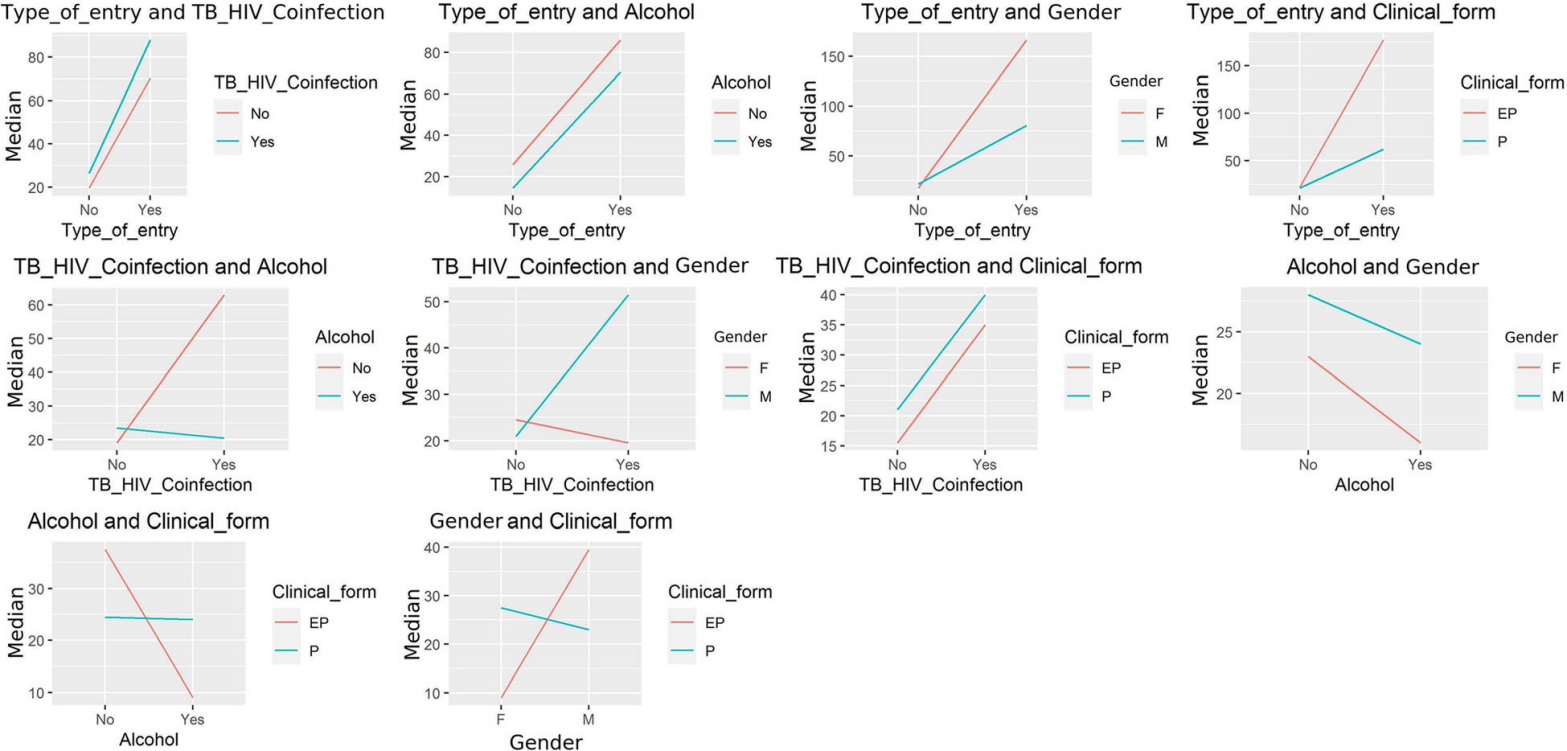
Interaction plots for each pair of explanatory variables according to survival time, Curitiba-Brazil

Figure 3 shows the interaction plots for each pair of explanatory variables according to survival time.

The results of the survival analysis are shown in Table 4. Before performing the model, we evaluated the multicollinearity of the potential explanatory variables. All the variables had a VIF value that ranged from 1.11 to 1.42, which meant the absence of multicollinearity among them. Through the analysis, some possible interactions between the explanatory variables (Supplementary Material 1) were identified in the study, specifically between TB/HIV coinfection and gender and between age and gender. These interactions were included in the Cox proportional hazards regression model.

**Table 4:** Results of the Cox proportional hazards regression model of patients who died from TB and associated factors, Curitiba-Brazil

Explanatory variables	Coefficient	Hazard ratio	95% CI	*P* value
Type of entry				
New case		1	1	
Relapse	−0.98	0.37	0.22-0.63	< 0.01
TB/HIV coinfection				
No		1	1	
Yes	0.43	1.54	0.74-3.20	0.24
Alcoholism				
No		1	1	
Yes	0.44	1.55	1.04-2.30	0.03
Gender				
Female		1	1	
Male	1.87	6.49	1.46-28.80	0.01
Age	0.02	1.02	0.99-1.04	0.08
Clinical form				
Extrapulmonary		1	1	
Pulmonary	0.01	1.01	0.67-1.52	0.97
TB/HIV coinfection and gender				
Without coinfection and female		1	1	
With coinfection and male	−1.14	0.32	0.13-0.78	0.01
Age and gender (male)	−0.03	0.97	0.94-0.99	0.03

The model indicates that the variables associated with TB death were cases of relapse (HR = 0.37, 95% CI = 0.22-0.63), alcoholism (HR = 1.55, 95% CI = 1.04-2.30), male patients (HR = 6.49, 95% CI = 1.03-2.68). Variables with interactions were TB/HIV coinfection and male patients as well as age and male patients.

Regarding the variables without interactions, TB deaths in relapse cases were 0.3 times higher than in new cases and the individuals classified as suffering from alcoholism showed 1.55 more risk for the mortality outcome in their treatment. When considering the explanatory variables that presented interactions, male individuals with TB/HIV coinfection had a risk of death that was 0.5 times lower than that for male individuals without TB/HIV coinfection. Female individuals with TB/HIV coinfection had a risk of death that was 1.54 times higher than that for female patients without TB/HIV coinfection, regardless of whether we considered people of the same age in these comparisons. Still, in this comparison of groups, when we examined only individuals without coinfection, male patients had a risk of death that was 6.49 times higher than that of female patients.

Regarding the interaction between age and gender, a reduction in the mortality rate in older individuals was observed, specifically when male patients were compared with female patients. In this sense, at the age of 30, men had a risk of death that was 2.74 higher than women, at the age of 40, the risk reduced to 2.05 and, at the age 65, it reached the value of 1.0. Thus, older men had the same chance of the treatment outcome under analysis as women.

To evaluate the validity of the Cox model assumptions, we analyzed the proportional hazard for the regression model with the chi-squared goodness of fit (Supplementary Material 2). In addition, the analysis of the residuals of the model (Supplementary Material 3) showed no violation of the Cox model assumptions and good suitability of the final model.

### Statistics from the mixed-effect model application

Regarding precocious deaths, Table 5 shows the results obtained from the mixed-effect model application. According to the findings, patients who were alcoholic had a chance of precocious death 17.93 times greater than those who were not. Additionally, the chance of precocious death within 60 days was 10.48 times greater than the chance of early death within 30 days. We also observed that relapse seemed to have a protective effect regarding death.

**Table 5:** Mixed-effect model of the associated factors with precocious deaths due tuberculosis, Curitiba-Brazil^a^

	Estimate	Std. error	Odds ratio	95% CI	***P*** value
**Intercept**	0.94	2.98			0.75
**Type of entry**					
Relapse	−4.71	1.85	<0.01	0.01-0.34	0.01**
**TB/HIV coinfection**					
Yes	−1.99	1.47	0.13	0.01-2.44	0.17
**Alcoholism**					
Yes	2.88	1.39	17.93	1.16-276.58	0.03**
**Gender**					
Male	−2.32	1.51	0.09	0.005-1.89	0.12
**Age**	004	0.04	1.04	0.95-1.14	0.34
**Clinical form**					
Pulmonary	0.63	1.39	1.87	0.12-28.99	0.65
**Diagnosis time (60 days)**	2.35	0.66	3.58	2.89-10.48	<0.01**

**p value <0.05

^a^Power of test—54.00% (95% CI 39.32; 68.19) based on 50 Monte Carlo simulation

## Discussion

We aimed to analyze patient survival time with TB, from diagnosis to death, precocious deaths and associated factors in southern Brazil. The findings show that the median survival from diagnosis to death among those who died from TB was 23.5 days, i.e., 67.8 days in new cases and 384.4 in relapses.

Regarding patient survival time, the findings show that most of the deaths occurred within 2 months. Some studies [11, 12, 32] have investigated the phenomenon of precocious death among patients with TB, and one such study observed similar results with a median survival time of 21 days in Korea [32]. Another study found that 19% of patients died within 7 days and 41% died within the first month after the start of treatment for TB [12]. Another study, undertaken in Africa that used the beginning of treatment as the zero point for estimating survival time, found a mean of 2 months in 53.3% among people who started their TB treatment, and, in this case, the mortality among HIV-positive people was higher than that among people who were HIV negative or whose HIV status was unknown [18].

Regarding associated factors with survival time for all patients and when the interactions were not considered, the TB deaths were associated with relapse, alcoholism, and male gender. When the variables TB/HIV coinfection and gender were considered together, we observed that male patients with coinfection presented a lower risk of TB death; on the other hand, female patients with TB/HIV had a higher risk of death, which we hypothesize as indicating inequality between the genders and which should be investigated through new studies.

When we analyzed only the cases of precocious deaths through mixed-effect models, patients who were alcoholic had a greater chance of dying precociously than those who were not, which has been evidenced in the literature because they tend to have lower compliance with treatment. However, a curious finding was that the chance of precocious death within 60 days was 10.48 times greater than the chance of early death within 30 days, which indicates a gap in the care cascade, specifically in the phase of intensive therapy coming to an end (provision for TB new cases), increasing the chances of patients abandoning treatment.

The findings also indicate that new cases tended to die more precociously when compared to relapse cases. One hypothesis is that, in Brazil, the health services have prioritized *directly observed treatment short course* (DOTS) in the intensive phase of treatment, mainly in settings with very poor and limited resources, which might explain the risk of death 60 days after diagnosis [33]. DOTS has been defined as a priority for patients with a relapse in these settings, since they can spread multidrug-resistant TB (MDR-TB) (resistance to the two main drugs used in treatment, rifampicin and isoniazid) and therefore the follow-up strategy must be systematic and rigorous, avoiding a potential epidemic of MDR-TB in vulnerable territories [34]. MDR-TB is a concern because of the high cost of the treatment, its toxicity and the poor outcomes with available therapies [35].

Regarding DOTS, in the city under study, the average coverage of DOTS was 55%, [36], which reinforces our hypotheses that the adoption of selective DOTS occurs for specific periods during treatment or in patients who have relapsed. The coverage of DOTS is low in Brazil, which might explain why the success of treatment is still lower than recommended by the World Health Organization, i.e., near 71% when the goal is higher than 85% [1]. In Brazil, of every 10 people who begin treatment, at least one abandons the use of medications, thereby increasing the risk of MDR-TB and death [37].

Regarding the influence of both alcohol consumption and TB mortality, some studies have demonstrated similar results. A study carried out in the same region as the present study indicated a problem with alcoholism, revealing an association between unsuccessful outcomes and this health condition; this finding confirms the importance of screening TB patients for alcohol consumption [38]. One meta-analysis [39] found that alcoholism was linked to a greater risk (RR 1.35, 95% CI, 1.09-1.68) of contracting TB when compared to those without this health condition. The consequences of alcoholism were also associated with TB, such as malnutrition, mental disease, use of other substances, and disrupted homeostatic mechanisms [40, 41]. Additionally, alcohol use disrupts the immune response, increasing susceptibility to respiratory diseases such as TB [11, 42]. Alcoholism can also contribute to loss of earnings, family disruption, interpersonal violence, low self-esteem, and stigmatization, aspects that are commonly identified among TB patients and that caused the authors to hypothesize about the reason for their death. Alcoholism may compromise access to treatment, heighten the risk of co-occurring health problems and perpetuate the cycles of poverty, alcohol use, and TB [43].

Another aspect is the global epidemiological data on TB, which have demonstrated a higher risk of death in men than in women [44, 45]; however, this study evidenced an intriguing and complex issue. Female patients with a diagnosis of TB/HIV coinfection had a higher risk of death; male patients with this condition had a lower risk. It is important to note that this comparison must be made in people of the same sex because, when comparing men and women with HIV, the risk remains higher among men.

TB and HIV are public health problems that have a synergistic effect on each other, leading to a higher risk of unfavorable treatment results [46]. We corroborate this finding, although we understand that these risks are not randomly distributed among men and women. Brazil has achieved interesting progress in terms of access to antiretroviral therapy for HIV patients, with a stabilization in incidence, a reduction in opportunistic diseases, and increased mean survival time [47]. However, studies have identified gender inequality, observing that women with low income and schooling, who had monogamous relationships throughout their lives, were at risk of a delayed diagnosis of HIV/AIDS [48]; this phenomenon was also observed in the USA, where female patients at risk of delayed HIV diagnosis were poorer, non-urban, and possibly exposed to HIV heterosexually [49], which may be associated with the findings of the present study. There is little evidence to explain this result regarding the gender difference from the individual perspective in terms of TB mortality, but the results may also be associated with a low CD4 count, not receiving antiretroviral therapy or undergoing cotrimoxazole prophylaxis therapy, being a female sex worker, being older, and being bedridden [50, 51].

We also found that young men are at higher risk of death than older men if we compared them with women of the same age. Some studies have demonstrated a significant excess of TB mortality occurring in the young adult population [45], but, as identified in our study, sex and age are intercepted and produce different risk effects for death, information that is of great relevance to conducting TB treatments.

The literature indicates that the efficacy of treatment is higher than 99.9%, even for patients with HIV [52, 53], but the present study revealed that the majority of patients died precociously. Beyond DOTS, there exists a lack of strategies for complete recovery of patients leading to a delay in hospital admission, a lack of suitable management of side effects, multiple morbidities (not assessed by this study), and not assessing the risk of failure. Offering social benefits such as basic food baskets, transportation vouchers, income generation programs, and school integration are very important and need to be implemented effectively to ensure the continuity of treatment, adherence, and better life conditions [11, 54].

Because of the alarming number of failures and deaths, the Brazilian health authorities have reinforced the importance of continuing treatment until the end, initiated in 2020 during the COVID-19 pandemic, an action on the Internet and social media aimed at people with TB with the goal of improving adherence and reducing the occurrence of failures and deaths, emphasizing inclusive DOTS for all cities [37]. The WebDOT platform has been developed and implemented in some cities [55] as a solution alternative for patient support and medication monitoring over the internet during the pandemic [23].

The difficulty in accessing services at the moment of symptom onset [32], especially in vulnerable groups or when health service providers are not qualified to recognize a cough as being a clinical sign of TB, should be borne in mind [24]. This result suggests the need for an attention model that gives a higher value to the active search for patients within territories, the tracking of TB among the population at large and regular appointments for patients living with HIV [23, 56, 57].

Brazil has a special protocol in place for monitoring the deaths that occur with a mention of TB as one of the causes, a protocol that, among other aims, seeks to investigate these patients’ individual health conditions and their access to health services. Additionally, this protocol is used for analyzing and correcting the information that appears in the different information systems used, namely, the SIM, SINAN, and TB site [57]. This is a strategic initiative to improve the qualification of the data; however, according to evidence from the present study, it is important to verify the phase during which the patient died in a stratified fashion, whether the case was being monitored by the health service and whether this happened in an early or late phase of treatment. This is important because, depending on the phase in which the patient died, actions also need to be modulated, as death makes us wonder whether measures and protocols have been implemented effectively to have an impact on TB context as well on the quality of care [58].

One of the limitations of this study is the small population studied and very heterogeneous; therefore, the findings are not generalizable to other contexts and population. Other limitations are the use of secondary data, which were entered into the form in advance, as there were gaps in form filling or missing information. Additionally, we did not have information regarding onset of clinical symptoms in patients, as this information was not available in the SINAN. The issue of alcohol consumption information is other bias, because the information is based on self-reports from patients or the subjective analysis of health workers, which might influence the findings; the culture in the region may stigmatize alcohol use in females, which was not verified in the present study.

The methodology used by the health services to confirm TB deaths, in most cases, did not use the death audit strategy or even verbal autopsy, which may be a difficult in the certification of deaths that occur in Brazil. Because of the restrictive information contained in the SIM and the SINAN, we encourage the development of new studies, mainly mixed methods research, to fill gaps that are not well-understood.

Since the SIM and the SINAN are not interconnected, it is difficult for health professionals and health managers to follow up on patients and monitor the evolution of their treatment, including the circumstances of death, if associated with TB. Therefore, this study contributes to advancing this issue in Brazil, as it is first to approach deaths due to TB and the integration/linkage of databases as a retrospective longitudinal study. Despite the study’s limitations from both systems, they are official sources and references to monitor and understand the TB situation in Brazil.

We emphasize that this study has made important contributions to understanding the real magnitude of death due to TB, since this is still underestimated. We used an original and specific method to link databases and then monitor patient survival time, and we also identified patients who died precociously due to TB. While these patients who died due to TB remain underestimated and undervalued, we never will manage and solve this problem in a proper and fair way.

## Conclusion

Most of the deaths occurred within 2 months after the diagnosis, during the intensive phase of the treatment. The use of alcohol and gender were associated with death, revealing inequality between men and women. We also observed that male patients with coinfection had a lower risk of precocious TB death and, conversely, female patients with comorbidity had a higher risk of early death. The findings also revealed that new cases tended to die more precociously when compared to relapse cases. The chance of precocious death within 60 days was 10.48 times greater than the chance of early death within 30 days. This study advanced knowledge regarding the identification of the vulnerabilities associated with mortality through a novel methodology. The findings must be addressed to fill a gap in the care cascades for active TB and ensure equity in health.

## Supplementary Information

**Additional file 1: Table 1.** Likelihood ratio test and interaction between the variables for analyzing associated factors with precocious deaths, Curitiba - Brazil.

**Additional file 2: Table 2.** Results for the test of proportional hazards assumption of the Cox regression model fit, Curitiba - Brazil.

**Additional file 3.** The analysis of the residuals of the model.

## Data Availability

The database is maintained by the Epidemiological Surveillance Division and Secretary of Health of the State of Parana, Brazil, and restrictions apply to the availability of these data, which were used under license for the current study as they are not publicly available. The first author had registered with details as well as contact data in the case of interest in collaborative work or further information.

## Abbreviations

DOTS: Directly observed treatment short-course
GAMLSS: Generalized additive model for location, scale, and shape
HDI: Human Development Index
HIV: Human immunodeficiency virus
MDR-TB: Multidrug-resistant tuberculosis
SIM: Mortality Information System
SINAN: Disease Notification Information System
TB: Tuberculosis
