# Supplementary material for: Survival time among patients who were diagnosed with tuberculosis, the precocious deaths and associated factors in southern Brazil
Source: Trop Med Health. 2021 Apr 21;49:31. doi: 10.1186/s41182-021-00320-4 (PMC8058757; doi:10.1186/s41182-021-00320-4)
Supplement: Supplementary file 1 — Additional file 1: Table 1. Likelihood ratio test and interaction between the variables for analyzing associated factors with precocious deaths, Curitiba - Brazil. [file 41182_2021_320_MOESM1_ESM.docx]

**Table 1** - Likelihood ratio test and interaction between the variables for analyzing associated factors with precocious deaths, Curitiba - Brazil.

| **Variables** | **Df** | **LogLik** | **Df_dif** | **Chisq** | **P.value** |
| --- | --- | --- | --- | --- | --- |
| Type_of_entry:Sex_H0 | 9 | -124.00 |  |  |  |
| Type_of_entry:Sex_H1 | 10 | -123.49 | 1 | 1.01 | 0.31 |
| Type_of_entry:Clinical_form_H0 | 9 | -124.00 |  |  |  |
| Type_of_entry:Clinical_form_H1 | 10 | -122.07 | 1 | 3.85 | 0.04 |
| TB_HIV_Coinfection:Alcohol_H0 | 9 | -124.00 |  |  |  |
| TB_HIV_Coinfection:Alcohol_H1 | 10 | -123.51 | 1 | 0.97 | 0.32 |
| TB_HIV_Coinfection:Sex_H0 | 9 | -124.00 |  |  |  |
| TB_HIV_Coinfection:Sex_H1 | 10 | -123.95 | 1 | 0.09 | 0.76 |
| Alcohol:Clinical_form_H0 | 9 | -124.00 |  |  |  |
| Alcohol:Clinical_form_H1 | 10 | -124.76 | 1 | 1.52 | 0.21 |
| Sex:Clinical_form_H0 | 9 | -124.00 |  |  |  |
| Sex:Clinical_form_H1 | 10 | -124.93 | 1 | 1.87 | 0.17 |
| Time:Type_of_entry_H0 | 9 | -124.00 |  |  |  |
| Time:Type_of_entry_H1 | 10 | -124.39 | 1 | 0.79 | 0.37 |
| Time:Clinical_form_H0 | 9 | -124.00 |  |  |  |
| Time:Clinical_form_H1 | 10 | -123.50 | 1 | 0.98 | 0.32 |
| Time:TB_HIV_Coinfection_H0 | 9 | -124.00 |  |  |  |
| Time:TB_HIV_Coinfection_H1 | 10 | -123.74 | 1 | 0.50 | 0.47 |
| Time:Alcohol_H0 | 9 | -124.00 |  |  |  |
| Time:Alcohol_H1 | 10 | -121.68 | 1 | 4.63 | 0.03 |
| Time:Sex_H0 | 9 | -124.00 |  |  |  |
| Time:Sex_H1 | 10 | -123.65 | 1 | 0.68 | 0.40 |
| Type_of_entry:Age_H0 | 9 | -124.00 |  |  |  |
| Type_of_entry:Age_H1 | 10 | -124.00 | 1 | 0.004 | 0.94 |
| TB_HIV_Coinfection:Age_H0 | 9 | -124.00 |  |  |  |
| TB_HIV_Coinfection:Age_H1 | 10 | -124.27 | 1 | 0.5406 | 0.4622 |
| Alcohol:Age_H0 | 9 | -124.00 |  |  |  |
| Alcohol:Age_H1 | 10 | -120.71 | 1 | 6.5654 | 0.0104 |
| Sex:Age_H0 | 9 | -124.00 |  |  |  |
| Sex:Age_H1 | 10 | -122.53 | 1 | 2.9348 | 0.0867 |
| Clinical_form:Age_H0 | 9 | -124.00 |  |  |  |
| Clinical_form:Age_H1 | 10 | -123.49 | 1 | 1.0036 | 0.3164 |
