# Supplementary material for: Survival time among patients who were diagnosed with tuberculosis, the precocious deaths and associated factors in southern Brazil
Source: Trop Med Health. 2021 Apr 21;49:31. doi: 10.1186/s41182-021-00320-4 (PMC8058757; doi:10.1186/s41182-021-00320-4)
Supplement: Supplementary file 2 — Additional file 2: Table 2. Results for the test of proportional hazards assumption of the Cox regression model fit, Curitiba - Brazil. [file 41182_2021_320_MOESM2_ESM.docx]

**Table 2 -** Results for the test of proportional hazards assumption of the Cox regression model fit, Curitiba - Brazil.

| Explanatory variables | Chi-squared | p-value |
| --- | --- | --- |
| Entry type | 0.6097 | 0.4349 |
| TB/HIV coinfection | 0.7491 | 0.3868 |
| Alcoholism | 1.4470 | 0.2290 |
| Gender | 0.0511 | 0.8211 |
| Age | 1.5614 | 0.2115 |
| Clinical form | 0.9039 | 0.3417 |
| TB/HIV coinfection and gender | 0.4240 | 0.5149 |
| Age and gender (male) | 0.9925 | 0.3191 |
| Global | 6.2962 | 0.6141 |
