# Supplementary figures and images for: Survival time among patients who were diagnosed with tuberculosis, the precocious deaths and associated factors in southern Brazil
Source: Trop Med Health. 2021 Apr 21;49:31. doi: 10.1186/s41182-021-00320-4 (PMC8058757; doi:10.1186/s41182-021-00320-4)

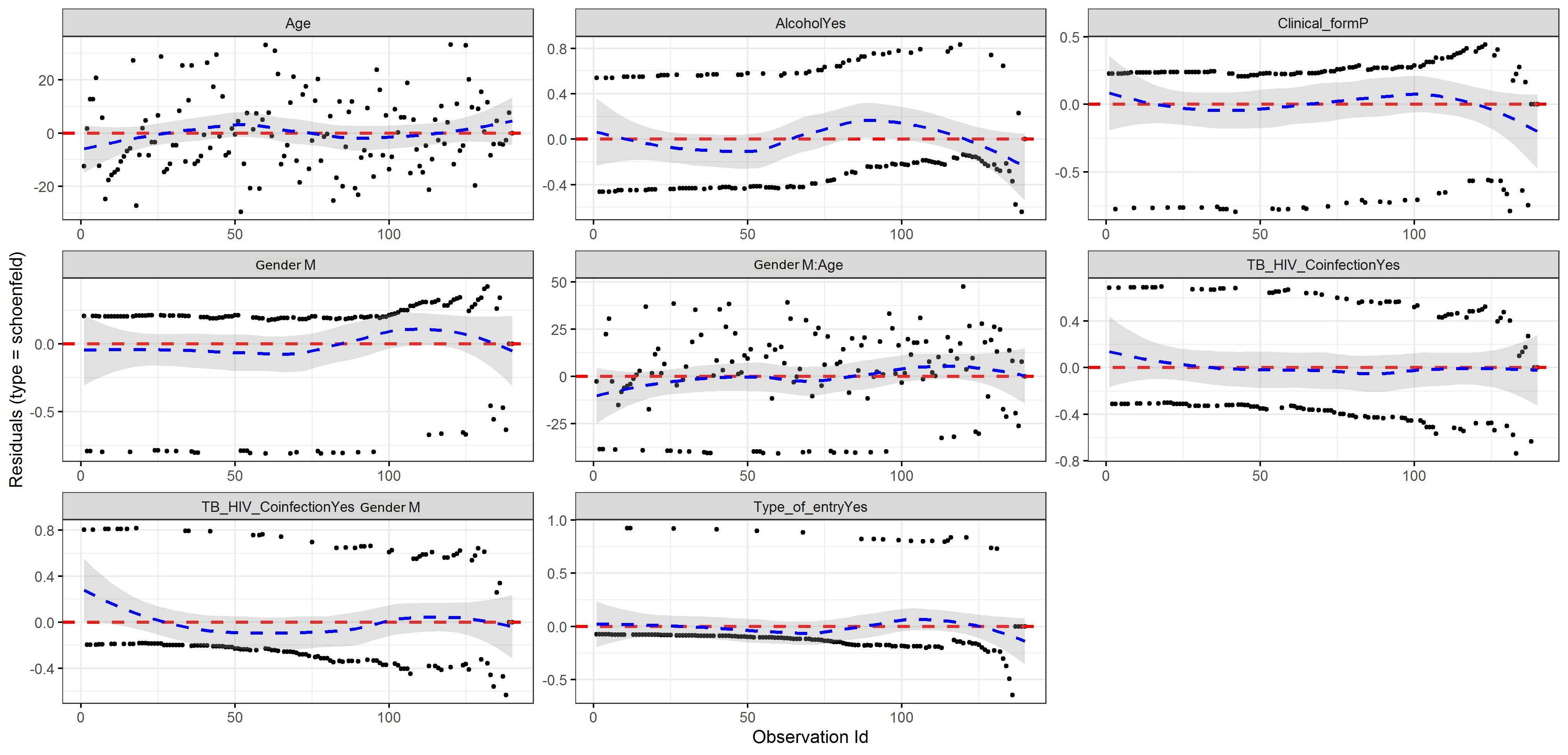

Supplement: Supplementary file 3 — Additional file 3. The analysis of the residuals of the model. [file 41182_2021_320_MOESM3_ESM.jpeg]
